# É possível universalizar os programas municipais de renda básica no Brasil? Uma estimação com dados de finanças municipais

**DOI:** 10.1590/0102-311XPT157325

**Published:** 2026-07-27

**Authors:** Vitória Alves Pereira Santos, Matías Mrejen

**Affiliations:** 1 Câmara Municipal do Rio de Janeiro, Rio de Janeiro, Brasil.; 2 Instituto René Rachou, Fundação Oswaldo Cruz, Belo Horizonte, Brasil.

**Keywords:** Política Social, Renda, Programas Sociais, Social Policy, Income, Social Programs, Política Social, Renta, Programas Sociales

## Abstract

Este estudo investiga a viabilidade fiscal da universalização de programas municipais de renda básica no Brasil, tomando como modelo os programas Renda Básica de Cidadania de Maricá (RBC-Maricá) e o programa Moeda Social Araribóia de Niterói (MSA-Niterói). Utilizando dados do Cadastro Único e das finanças municipais de 5.570 municípios brasileiros, avaliamos qual seria o custo desses programas como proporção das despesas totais e sociais. Os resultados revelam que, para a maioria dos municípios, a implantação de programas nos moldes do RBC-Maricá e do MAS-Niterói seria significativamente mais onerosa do que para as cidades de referência. Observou-se uma acentuada desigualdade regional na capacidade de implantação, com as regiões Norte e Nordeste enfrentando um ônus fiscal excessivo, e o Sul e o Sudeste demonstram maior viabilidade, especialmente para o modelo MSA-Niterói. Ao simular cenários com esforços fiscais similares aos de Maricá e de Niterói, constatou-se um *trade-off* entre cobertura e valor do benefício, assim como desigualdades regionais muito grandes no valor das transferências. Conclui-se que a universalização de programas de renda básica no Brasil com base exclusivamente em recursos municipais é pouco provável devido às profundas disparidades regionais e ao elevado custo fiscal. O estudo sugere a necessidade de um modelo de financiamento que transcenda a esfera municipal, com substancial apoio do Governo Federal, e que esteja atrelado a um debate mais amplo sobre justiça fiscal e redistribuição de renda para garantir a equidade na distribuição dos benefícios e o bem-estar da população.

## Abstract (en)

This study investigates the fiscal feasibility of universalizing municipal basic income programs in Brazil by taking the Basic Citizenship Income program in Maricá and Araribóia Social Currency program in Niterói as models. Using data from the Unified Registry and the finances of 5,570 Brazilian municipalities, we estimated the cost of these programs as a proportion of total and social expenditures. The results show that, for most municipalities, the implementation of programs along the lines of the studied models would be significantly more costly than for the reference municipalities. This study observed a marked regional inequality in implementation capacity, with the Brazilian North and Northeast facing an excessive fiscal burden, whereas its South and Southeast showed greater viability, especially for the Niterói model. Simulating scenarios with fiscal efforts resembling those in Maricá and Niterói found a trade-off between coverage and benefit and very large regional inequalities in transfer value. The universalization of basic income programs in Brazil based exclusively on municipal resources is unlikely due to deep regional disparities and high fiscal cost. This study suggests the need for a financing model that goes beyond the municipal sphere, enjoying substantial federal support and a link to a broader debate on fiscal justice and income redistribution to ensure equal benefit distribution and the population’s well-being.

## Abstract (es)

Este estudio evalúa la viabilidad fiscal de la universalización de los programas municipales de renta básica en Brasil a partir del modelo de los programas Renta Básica de Ciudadanía de Maricá (RBC-Maricá) y Moneda Social Araribóia de Niterói (MSA-Niterói). Con base en datos del Registro Único y de las finanzas municipales de 5.570 municipios brasileños, se evaluó el costo de estos programas como una proporción de los gastos total y social. Los resultados revelan que la mayoría de los municipios tenían significativamente una implementación de los programas en los ámbitos de RBC-Maricá y MSA-Niterói más costosa que para las ciudades de referencia. Hubo una marcada desigualdad regional en la capacidad de implementación, con las regiones Norte y Nordeste enfrentando una carga fiscal excesiva, mientras que el Sur y Sudeste demuestran una mayor viabilidad, especialmente para el modelo MSA-Niterói. Al simular escenarios con esfuerzos fiscales similares a los de Maricá y Niterói, se observó un *trade-off* entre la cobertura y el valor de los beneficios, así como grandes desigualdades regionales en el valor de las transferencias. Se concluye que la universalización de los programas de renta básica en Brasil basados exclusivamente en recursos municipales es poco viable a causa de las profundas disparidades regionales y del alto costo fiscal. Este estudio apunta la necesidad de un modelo de financiamiento que supere el ámbito municipal, con un apoyo clave del Gobierno Federal, y que esté vinculado a un debate más amplio acerca de la justicia tributaria y de la redistribución del ingreso para garantizar la equidad en la distribución de los beneficios y el bienestar de la población.

## Introdução

As transferências de renda têm sido um instrumento recorrente ao longo da história para enfrentar a pobreza, a instabilidade social e as transições econômicas. A história oferece evidências concretas sobre a eficácia e a adaptabilidade delas como uma intervenção estratégica capaz de empoderar indivíduos, estimular as economias locais e contribuir para a estabilidade social e política ^1^.

Alguns dos debates centrais ao redor dessas políticas têm se concentrado na escolha entre assistência condicional ou incondicional e nos métodos para a seleção (ou focalização) de beneficiários. As transferências condicionadas estão associadas ao cumprimento de requisitos específicos, como a frequência escolar das crianças, a realização de consultas de pré-natal ou a vacinação de crianças, sendo vistas como instrumentos capazes de promover simultaneamente a redução da pobreza e o investimento em capital humano ^2^. Já as transferências sem condicionamentos não impõem contrapartidas formais aos beneficiários, partindo do princípio de respeito à autonomia individual e da confiança de que os indivíduos são os melhores juízes de suas próprias necessidades ^3^ ^,^ ^4^.

No que tange à seleção de beneficiários, os programas podem ser focalizados, destinando os recursos a grupos específicos que atendem a determinados critérios (como linhas de pobreza ou vulnerabilidade demográfica), priorizando beneficiar os setores da população com menor renda ou maior vulnerabilidade socioeconômica. Alternativamente, podem seguir o princípio da universalidade, em que todos os membros de uma comunidade política são elegíveis, tratando o benefício como um direito de cidadania e priorizando evitar erros de exclusão e a estigmatização dos beneficiários ^5^ ^,^ ^6^.

Outros temas relevantes dizem respeito à regularidade, à individualidade e à forma de provisão do benefício. A regularidade refere-se à previsibilidade temporal das transferências, isto é, à garantia de que os pagamentos ocorram de forma contínua e estável, o que é essencial para o planejamento familiar e a segurança de renda. Já a individualidade diz respeito à titularidade e ao cômputo do benefício: se ele é concedido à unidade familiar como um todo, por intermédio de um chefe de família e sem computar o mesmo benefício por cada indivíduo, ou computando de forma individual o valor dos benefícios. Por fim, a forma de transferência - se em dinheiro, em espécie ou por meio de cartões ou *vouchers* com restrições de uso - também tem sido objeto de debate. As transferências em dinheiro conferem maior liberdade de escolha aos beneficiários e costumam gerar ganhos de bem-estar e eficiência ao permitir a alocação conforme as necessidades individuais ^3^ ^,^ ^7^. Em contraste, transferências em espécie ou com limitações de consumo - como vales-alimentação ou cartões destinados exclusivamente à compra de determinados bens - buscam assegurar que os recursos sejam aplicados em itens considerados socialmente prioritários, embora possam reduzir a autonomia dos beneficiários e aumentar custos administrativos ^7^ ^,^ ^8^.

As diferentes combinações de condicionalidade, focalização, regularidade, individualidade e forma de provisão do benefício permitem delinear diferentes desenhos de políticas de transferência de renda. Focando em políticas que são transferidas de forma regular e em dinheiro, mas que variam nas dimensões de focalização e condicionalidade, podemos pensar em três programas diferentes: transferências condicionadas de renda, renda básica universal e renda básica.

Os programas de transferência condicionada de renda são provavelmente o modelo mais difundido e estudado. Esses programas, como o Programa Bolsa Família no Brasil ou o Progresa/Oportunidades no México, consistem em repasses monetários a famílias em situação de pobreza ou vulnerabilidade socioeconômica, condicionados ao cumprimento de obrigações associadas à saúde e educação, frequentemente para as crianças do grupo familiar. A literatura documenta extensamente seus impactos positivos sobre a redução da pobreza, a melhoria de indicadores educacionais e nutricionais e o fortalecimento da autonomia das mulheres, embora também existam críticas quanto à complexidade administrativa e à dependência das condicionalidades ^2^ ^,^ ^9^ ^,^ ^10^. No Brasil, por exemplo, há ampla evidência sobre os benefícios do Programa Bolsa Família para a saúde da população ^11^ ^,^ ^12^ ^,^ ^13^.

A renda básica universal, por sua vez, propõe o pagamento incondicional de um valor monetário a todos os cidadãos, independentemente da renda ou da condição de trabalho. Defensores dessa proposta, como Philippe Van Parijs, argumentam que ela representa uma forma de redistribuição compatível com a liberdade individual e a justiça social, ao assegurar a todos um piso de segurança econômica ^14^ ^,^ ^15^. Contudo, a maioria das experiências de renda universal foram programas piloto - como os testes realizados na Finlândia e em alguns municípios europeus - e os desafios fiscais e políticos de sua implantação em larga escala permanecem significativos ^16^ ^,^ ^17^.

Entre esses extremos, os programas de renda básica ou garantida podem adotar critérios mais ou menos amplos de elegibilidade, priorizando maior focalização nos mais pobres ou vulneráveis, ou priorizando se aproximar da universalidade, mas sem incorporar condicionalidades. A evidência sugere que esses arranjos têm potencial de afetar positivamente a saúde mental e o bem-estar dos adultos ^18^ e a saúde e educação das crianças ^19^.

Considerando esse potencial, neste trabalho, nós avaliamos as perspectivas para estender a cobertura de programas de renda básica no Brasil nos moldes de dois dos programas de renda básica de maior envergadura no país: o Renda Básica de Cidadania de Maricá (RBC-Maricá) e o Moeda Social Araribóia de Niterói (MSA-Niterói), ambos no Estado do Rio de Janeiro. Mais especificamente, nós avaliamos: (a) qual seria o custo como proporção das despesas totais e como proporção das despesas sociais (assistência social, previdência social, saúde e educação) para os municípios brasileiros se eles implantassem esses programas; e (b) qual seria a transferência *per capita* média realizada por programas com os critérios de elegibilidade do RBC-Maricá e do MSA-Niterói, se eles demandam o mesmo percentual das despesas totais do município do que esses programas demandam em Maricá e Niterói, respectivamente.

Compreender as perspectivas para estender programas de renda básica em nível nacional resulta relevante. No Brasil, um marco para a implantação de programas de renda básica foi a sanção da *Lei nº 10.835/2004* ^20^, proposta pelo então senador Eduardo Suplicy, que institui a “*renda básica de cidadania*” no país. A lei determinou que, a partir de 2005, todos os brasileiros residentes no país e estrangeiros residentes há pelo menos cinco anos teriam o direito de receber um benefício monetário, independentemente de sua condição socioeconômica, com valor “*suficiente para atender às despesas mínimas de cada pessoa com alimentação, educação e saúde, considerando para isso o grau de desenvolvimento do país e as possibilidades orçamentárias*” ^20^. No entanto, a implantação da Renda Básica de Cidadania em nível nacional no Brasil não foi concretizada e foram privilegiados outros programas de transferência de renda, como o Programa Bolsa Família (focado em famílias em situação de pobreza e extrema pobreza, e que possui condicionalidades) e o Benefício de Prestação Continuado (focado em idosos de baixa renda e pessoas com alguma deficiência).

Em anos recentes, programas municipais de renda básica de cidadania, financiados com *royalties* do petróleo, surgiram no país. Uma das principais referências é o programa RBC-Maricá, surgido em 2015 como um valor complementar pago no município a beneficiários do Programa Bolsa Família. O programa teve como antecedente o Programa Municipal de Economia Solidária, Combate à Pobreza e Desenvolvimento Econômico e Social de Maricá, criado em 2013, responsável pela criação do Banco Comunitário e da moeda social Mumbuca, desde então usada para programas municipais de transferência de renda ^21^. Em 2023, o programa constava de uma transferência de 200 Mumbucas (uma moeda de circulação local com paridade com o Real brasileiro) para o responsável de cada grupo familiar, acrescentado de 200 Mumbucas por cada membro adicional do domicílio, sem limite máximo de transferências por famílias. Para ser beneficiário, a renda familiar mensal tem de ser inferior a três salários mínimos ^22^ ^,^ ^23^. Outra iniciativa relevante é o programa Moeda Social Araribóia de Niterói, implantado em janeiro de 2022. Em 2023, o programa constava de uma transferência de 250 Araribóias (uma moeda de circulação local com paridade com o Real brasileiro) para o responsável de cada grupo familiar, acrescentado de 90 Araribóias por cada membro adicional do domicílio, até um limite de 700 Araribóias (ou seja, até cinco membros adicionais do domicílio). Para ser beneficiário, tem de estar em situação de vulnerabilidade social e residir na cidade de Niterói ^24^.

Esses programas podem ser caracterizados como programas de renda básica: o pagamento é feito regularmente todo mês, conforme valores fixos e predeterminados e em unidades monetárias que, apesar de terem circulação restrita aos municípios, podem ser usadas livremente dentro destes. Esse desenho baseado no uso de moedas locais pode ser visto como um comprometimento para a liberdade individual do beneficiário, pois limita a capacidade de consumo fora do município, mas existem evidências do seu efeito positivo na questão fiscal, por incentivar a economia local ^25^. Observe-se que a regulação das moedas sociais é atualmente objeto de debate no Congresso Nacional ^26^. Adicionalmente, os programas não demandam contrapartidas nem condicionalidades dos beneficiários e são acumuláveis com rendimentos de qualquer natureza. O RBC-Maricá, por sua vez, também respeita parcialmente o princípio da individualidade, já que as transferências são computadas de forma individual (embora a elegibilidade seja determinada no nível do grupo familiar) ^27^.

A discussão sobre programas municipais de renda básica no Brasil é frequentemente ilustrada pelos casos de Maricá e Niterói, apesar de existirem outros casos como os de Indiaroba (Sergipe) e Porciúncula (Rio de Janeiro). Contudo, a replicabilidade dessas experiências é questionável dadas as características singulares desses municípios. Niterói, por exemplo, é o quinto município do país com a maior renda familiar *per capita* média e concentra 5% do total de *royalties* repassados pela União a municípios, apesar de contar com somente 0,2% da população do país. Maricá, por sua vez, tem o nono maior produto interno bruto (PIB) *per capita* e recebe 13% do total desses *royalties*, apesar de contar com somente 0,1% da população brasileira (cálculos próprios com base em dados do Instituto Brasileiro de Geografia e Estatística e da Agência Nacional do Petróleo, Gás Natural e Biocombustíveis). Em 2023, a receita dos *royalties* representou 37% das receitas correntes de Maricá e 15% das de Niterói (cálculos próprios baseando-se em dados do Sistema de Informações Contábeis e Fiscais do Setor Público Brasileiro - SICONFI, do Ministério da Fazenda). Dada essa realidade excepcional, a simples extrapolação desses modelos para outros municípios pode não ser adequada. Portanto, o objetivo deste artigo é analisar o impacto fiscal de programas de renda básica para as finanças municipais, propondo um quadro de análise que possa ser utilizado para avaliar a viabilidade da implantação de políticas semelhantes em diferentes contextos municipais brasileiros.

## Métodos

### Bases de dados

Para avaliar o potencial impacto fiscal da implantação dos programas RBC-Maricá e MSA-Niterói em outros municípios, utilizamos informações publicamente disponíveis sobre a população inscrita no Cadastro Único (CadÚnico) nos 5.570 municípios brasileiros, disponibilizadas pela Secretaria de Avaliação, Gestão da Informação e Cadastro Único do Ministério do Desenvolvimento e Assistência Social, Família e Combate à Fome ^28^. Inicialmente, utilizamos informações que detalham o número de famílias e de indivíduos cadastrados no CadÚnico por faixa de renda em cada município, para determinar o número de famílias e o valor dos benefícios que seriam transferidos por cada um dos programas, se implantados em cada município. Consideramos os dados disponíveis até fevereiro de 2023, por se tratar do recorte mais recente com detalhamento suficiente sobre o perfil socioeconômico das famílias no momento do processamento dos dados. O Quadro 1 resume as características dos programas municipais de renda básica consideradas para o cálculo do valor da transferência para cada família.

**Quadro 1:** Características principais dos programas de renda básica de Maricá e de Niterói, Rio de Janeiro, Brasil, fevereiro de 2023.

	Renda Básica de Cidadania de Maricá (RBC-Maricá)	Moeda Social Araribóia de Niterói (MSA-Niterói)
Paridade	1,00 Mumbuca = R$ 1,00	1,00 Araribóia = R$ 1,00
Valor do benefício por responsável familiar	R$ 200,00	R$ 250,00
Valor adicional	R$ 200,00	R$ 90,00
Valor máximo das transferências por família	Não há	R$ 700,00 (cobertura de até 5 membros adicionais)
Critérios de elegibilidade	Ter renda familiar mensal de até 3 salários mínimos e residir na cidade há pelo menos 3 anos	Estar em situação de vulnerabilidade social e residir na cidade

Fonte: elaboração própria.

Utilizamos também dados do SICONFI sobre as despesas empenhadas pelos governos municipais no ano de 2023, tanto o total quanto a soma das áreas sociais (assistência, previdência, saúde e educação), para ver qual o percentual dessas despesas demandaria cada programa ^29^.

### Cálculo do gasto com programas de renda básica por município

#### Modelo Renda Básica de Cidadania de Maricá

Para o modelo RBC-Maricá, identificamos o número de indivíduos membros de famílias inscritas no CadÚnico residentes em cada município com renda familiar mensal de até três salários mínimos, sem considerar um tempo mínimo de residência por não possuirmos esta informação. Calculamos o valor anualizado para 12 meses, considerando uma transferência mensal equivalente a R$ 200,00 por indivíduo, seguindo a seguinte fórmula:

$${RBC}_{m}=\;12\times 200\times M_{m}$$

Em que *RBC*_*m*_ representa o gasto estimado com o programa segundo o modelo RBC-Maricá no município *m*, *M*_*m*_ representa o número de membros de famílias inscritas no CadÚnico com renda mensal de até três salários mínimos no município. Nesse sentido, *M*_*m*_ inclui todos os responsáveis e membros adicionais das famílias que cumprem o critério de elegibilidade.

Usando os dados de despesas empenhadas pelos municípios, calculamos quanto *RBC*_*m*_ representa como percentual das despesas totais e das despesas sociais de cada município. Apresentamos os resultados agrupados em cinco faixas: ≤ 5%; > 5% a ≤ 10%; > 10% a ≤ 15%; > 15% a ≤ 20%; e > 20%. Interpretamos essa medida como um indicador da viabilidade fiscal da implantação de um programa nos moldes do RBC-Maricá em cada município. Mostramos também para cada região geográfica (Norte, Nordeste, Sudeste, Centro-oeste, Sul) o percentual de municípios em cada uma dessas faixas.

Alternativamente, calculamos qual seria a transferência média por beneficiário e o percentual da população coberta se cada município dedicasse a um programa de renda básica o mesmo percentual das despesas sociais que o Município de Maricá dedica (11,2%) e um critério de elegibilidade similar (uma transferência por cada membro de famílias inscritas no CadÚnico com renda de até três salários mínimos). Reportamos as médias para cada Unidade da Federação (UF). Interpretamos essa medida como uma estimação de quão desiguais seriam os programas de renda básica financiados pelos municípios se eles fizessem o mesmo esforço fiscal que Maricá e adotassem um programa de renda básica priorizando maximizar sua cobertura, se aproximando mais da universalização.

#### Modelo Moeda Social Araribóia de Niterói

Para o modelo MSA-Niterói, identificamos o número de indivíduos membros de famílias em situação de vulnerabilidade social (pobreza ou extrema pobreza) inscritas no CadÚnico residentes em cada município. Calculamos o valor anualizado para 12 meses, seguindo a seguinte fórmula:

$${MSA}_{m}=12\times min\;\left\{\left[250V_{m}+90\left(A_{m}-V_{m}\right)\right];\;700V_{m}\right\}$$

Em que *MSA*_*m*_ representa o gasto estimado com o programa segundo o modelo MSA-Niterói no município *m*, *A*_*m*_ representa o número de indivíduos em situação de vulnerabilidade social e *V*_*m*_ o número de famílias em situação de vulnerabilidade social. Ou seja, em municípios com uma média de até cinco indivíduos por família em situação de vulnerabilidade social, o benefício médio considera R$ 250,00 por responsável mais R$ 90,00 por membro adicional. Em municípios com uma média maior que cinco indivíduos por família em situação de vulnerabilidade social, o benefício médio é limitado em R$ 700,00 por família. Na implantação do programa, esse limite é considerado no nível da família, mas não foi possível fazer o cálculo dessa forma por trabalharmos com dados agregados ao nível do município.

Usando os dados de despesas empenhadas pelos municípios, calculamos quanto *MSA*_*m*_ representa como percentual das despesas totais e das despesas sociais de cada município. Interpretamos essa medida como um indicador da viabilidade fiscal da implantação de um programa nos moldes do MSA-Niterói em cada município. Apresentamos os resultados agrupados em cinco faixas: ≤ 5%; > 5% a ≤ 10%; > 10% a ≤ 15%; > 15% a ≤ 20%; e > 20%. Mostramos também para cada região geográfica o percentual de municípios em cada uma dessas faixas.

Alternativamente, calculamos qual seria a transferência média por beneficiário e o percentual da população coberta se cada município dedicasse a um programa de renda básica o mesmo percentual das despesas sociais que o Município de Niterói dedica (9,2%) e um critério de elegibilidade similar (uma transferência por cada membro de famílias em situação de vulnerabilidade social). Reportamos as médias para cada UF. Interpretamos essa medida como uma estimação de quão desiguais seriam os programas de renda básica financiados pelos municípios se eles fizessem o mesmo esforço fiscal que Niterói, e adotassem um programa de renda básica priorizando a focalização desta na população em situação de vulnerabilidade social.

## Resultados

**Figura 1:**
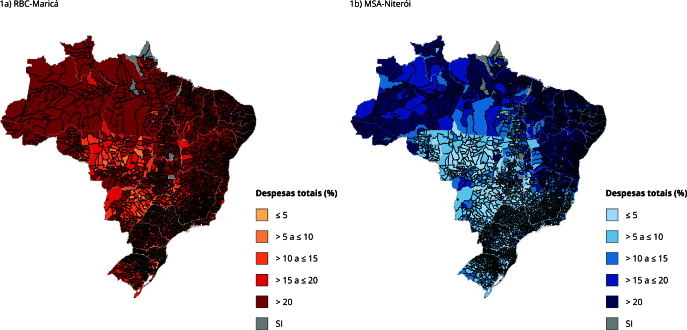
Gasto com programas de renda básica segundo percentual das despesas totais do município.

Começamos olhando para a viabilidade de implantação de programas municipais de renda básica inspirados no RBC-Maricá e no MSA-Niterói, analisando o impacto que estes teriam nas despesas dos municípios brasileiros. A Figura 1 mostra os resultados considerando quanto esses programas representariam como percentual das despesas correntes empenhadas em cada município em 2023. Os resultados sugerem que programas municipais de renda básica seriam bastante onerosos para a ampla maioria dos municípios brasileiros. Enquanto o custo dos seus programas estimado para Maricá e Niterói é inferior a 5% da despesa total, em cada caso, a média do percentual das despesas totais dedicado aos programas seria marcadamente maior nos demais municípios: 26,9% para programas nos moldes do RBC-Maricá e 12,9% para programas nos moldes do MSA-Niterói. Ou seja, os municípios dedicariam, na média, uma proporção dos seus recursos mais do que cinco vezes maior do que a de Maricá para implantar o RBC-Maricá e quase três vezes maior do que a de Niterói para implantar o MSA-Niterói. Analogamente, em 98,1% dos municípios o gasto com o RBC-Maricá superaria 5% das despesas totais - em 75% superaria este patamar com o MSA-Niterói.

MSA-Niterói: Moeda Social Araribóia de Niterói; RBC-Maricá: Renda Básica de Cidadania de Maricá; SI: sem informação. Nota: os mapas mostram o percentual da despesa total empenhada dos municípios que demandariam implantar programas similares ao RBC-Maricá (à esquerda) e ao MSA-Niterói (à direita).

**Figura 2:**
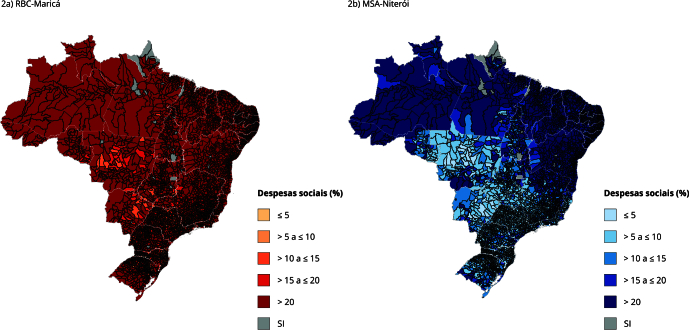
Gasto com programas de renda básica segundo porcentual das despesas sociais do município.

A Figura 2 mostra os resultados considerando quanto esses programas representariam como percentual das despesas sociais em cada município em 2023. Novamente, os programas comprometeriam parcelas muito maiores das despesas sociais do que as empenhadas em 2023 por Maricá (11,2%) e Niterói (9,2%) com seus respectivos programas. O RBC-Maricá demandaria, na média, 38,7% da despesa dos municípios com saúde, educação, assistência social e previdência, e o MSA-Niterói demandaria uma média de 15,2%. Analogamente, o RBC-Maricá demandaria mais de 20% da despesa social em 80% dos municípios brasileiros e o MSA-Niterói em 39,8% dos municípios.

MSA-Niterói: Moeda Social Araribóia de Niterói; RBC-Maricá: Renda Básica de Cidadania de Maricá; SI: sem informação. Nota: os mapas mostram o percentual da despesa social empenhada dos municípios que demandariam implantar programas similares ao RBC-Maricá (à esquerda) e ao MSA-Niterói (à direita). Foram consideradas como despesa social todas as despesas dos municípios com saúde, educação, assistência social e previdência.

A Tabela 1 mostra a distribuição dos municípios por região segundo a faixa de despesas totais e de despesas sociais que dedicariam a um programa com as características do RBC-Maricá. Os resultados sugerem que esse programa seria muito caro para a grande maioria dos municípios em todas as regiões. Enquanto Maricá gasta menos de 5% das suas despesas totais com as transferências do programa, nenhum município nas regiões Centro-oeste, Nordeste e Norte conseguiria dedicar uma parcela similar das suas despesas totais para um programa dessas características. Mesmo no Sul (7,2%) e no Sudeste (1,1%), a proporção de municípios que conseguiriam implantar programas desse tipo fazendo um esforço fiscal similar ao de Maricá é pequena. Adicionalmente, os resultados mostram que a distribuição regional do esforço fiscal que demandaria o RBC-Maricá para os municípios é desigual: somente no Sul (28,8%) e no Sudeste (8,4%) parcelas relevantes dos municípios conseguiriam implantar o RBC-Maricá dedicando menos que 15% da despesa social (comparável ao esforço de 11,2% da despesa social feito por Maricá).

**Tabela 1:** Distribuição dos municípios segundo gasto com o programa Renda Básica de Cidadania de Maricá (RBC-Maricá) como percentual das despesas do município (por região).

	Centro-oeste (%)	Nordeste (%)	Norte (%)	Sudeste (%)	Sul (%)
Despesas totais (%)					
≤ 5	0,0	0,0	0,0	1,1	7,2
> 5 a ≤ 10	4,5	0,1	0,4	8,8	26,4
> 10 a ≤ 15	23,1	0,5	1,8	23,1	24,3
> 15 a ≤ 20	25,5	2,1	6,0	20,9	19,8
> 20	44,8	96,3	86,7	45,8	21,7
Sem informação	2,1	1,1	5,1	0,4	0,5
Despesas sociais (%)					
≤ 5	0,0	0,0	0,0	0,2	2,1
> 5 a ≤ 10	0,0	0,0	0,0	1,7	9,3
> 10 a ≤ 15	3,6	0,1	0,2	6,5	17,4
> 15 a ≤ 20	9,9	0,1	1,1	14,9	17,0
> 20	84,2	98,7	93,6	76,3	53,7
Sem informação	2,4	1,2	5,1	0,4	0,5

Nota: a tabela mostra a distribuição dos municípios em cada região segundo faixas do percentual da despesa total e da despesa social empenhada que demandaria implantar um programa similar ao RBC-Maricá.

A Tabela 2 mostra resultados similares para programas com as características do MSA-Niterói. Os resultados sugerem que parcelas maiores dos municípios estariam em condições de implantar programas com essas características. O custo do programa equivaleria a menos de 5% das despesas totais (similar ao esforço realizado por Niterói) para a maioria dos municípios no Sul (61,7%) e para mais de um quarto dos municípios no Centro-oeste (29,8%) e Sudeste (29,6%). Ao mesmo tempo, as desigualdades regionais são muito acentuadas: em 87,8% dos municípios no Nordeste e 61,1% no Norte o MSA-Niterói demandaria mais do que 20% da despesa social, mais do que o dobro da parcela das despesas sociais dedicadas pela Prefeitura de Niterói ao programa, já essa proporção seria de 13,1% no Centro-oeste, 16,3% no Sudeste e 2,9% no Sul.

**Tabela 2:** Distribuição dos municípios segundo gasto com o programa Moeda Social Araribóia de Niterói (MSA-Niterói) como percentual das despesas do município (por região).

	Centro-oeste (%)	Nordeste (%)	Norte (%)	Sudeste (%)	Sul (%)
Despesas totais (%)					
≤ 5	29,8	0,1	4,4	29,6	61,7
> 5 a ≤ 10	45,4	2,7	16,4	40,2	29,8
> 10 a ≤ 15	14,6	11,0	16,9	17,0	6,9
> 15 a ≤ 20	5,8	25,6	16,9	7,7	0,8
> 20	2,4	59,6	40,2	5,2	0,3
Sem informação	2,1	1,1	5,1	0,4	0,5
Despesas sociais (%)					
≤ 5	8,6	0,0	0,4	11,3	38,0
> 5 a ≤ 10	34,7	0,3	6,4	36,2	35,1
> 10 a ≤ 15	28,3	2,1	12,0	23,4	17,5
> 15 a ≤ 20	13,1	8,5	14,9	12,4	5,9
> 20	13,1	87,8	61,1	16,3	2,9
Sem informação	2,4	1,2	5,1	0,4	0,5

Nota: a tabela mostra a distribuição dos municípios em cada região segundo faixas do percentual da despesa total e da despesa social empenhada que demandaria implantar um programa similar ao MSA-Niterói.

Em conjunto, os resultados das Tabelas 1 e 2 sugerem que os programas RBC-Maricá e MSA-Niterói demandariam esforços fiscais maiores do que os feitos por Maricá e por Niterói para a maioria dos municípios se eles implantassem algum programa municipal de renda básica similar. Somente na Região Sul, o RSA-Niterói poderia ser implantado pela maioria dos municípios com esforços fiscais similares aos realizados por Niterói.

Nesse cenário, uma alternativa é avaliar o que aconteceria se os municípios fizessem um esforço fiscal comparável ao realizado por Maricá e por Niterói, dedicando parcelas similares de sua despesa social a programas municipais de renda básica. A Tabela 3 mostra os resultados desse exercício, considerando o que aconteceria com a cobertura dos programas e o valor transferido por beneficiário em dois cenários. No primeiro, os municípios dedicam uma parcela das despesas sociais similar à de Maricá (11,2%) para implantar um programa de renda básica, maximizando sua cobertura e se aproximando mais da universalização dele, adotando o critério de elegibilidade de todos os indivíduos em famílias com renda de até três salários mínimos. No segundo, os municípios dedicam uma parcela das despesas sociais similar à de Niterói (9,2%) para implantar um programa de renda básica priorizando a focalização, adotando o critério de elegibilidade de indivíduos em famílias em situação de vulnerabilidade social (pobreza ou extrema pobreza). Em todos os casos, são reportadas as médias considerando-se todos os municípios de cada UF ou região.

**Tabela 3:** Distribuição dos municípios segundo gasto com o programa Moeda Social Araribóia de Niterói (MSA-Niterói) como percentual das despesas do município (por região).

Região/UF	Priorizando maximizar a cobertura		Priorizando focalização	
	Cobertura (%)	Valor por beneficiário (R$)	Cobertura (%)	Valor por beneficiário (R$)
Norte	71,4	48,10	51,1	64,00
Acre	76,7	33,30	62,4	34,00
Amazonas	76,7	42,10	64,9	41,00
Amapá	80,8	48,70	69,3	47,00
Pará	72,3	43,70	58,0	47,00
Rondônia	53,9	65,70	24,8	128,00
Roraima	68,7	43,00	57,4	44,00
Tocantins	72,9	51,50	43,0	77,00
Nordeste	77,4	43,70	57,8	49,00
Alagoas	77,7	58,100	61,6	61,00
Bahia	76,3	40,00	55,9	45,00
Ceará	75,4	42,80	52,4	51,00
Maranhão	77,5	44,70	63,2	46,00
Paraíba	77,9	47,50	58,1	53,00
Pernambuco	75,8	37,90	58,0	41,00
Piauí	81,3	44,70	62,5	48,00
Rio Grande do Norte	78,4	45,40	52,4	58,00
Sergipe	76,6	41,80	58,0	46,00
Centro-oeste	54,6	79,60	28,7	143,00
Distrito Federal	26,7	-	17,5	-
Goiás	56,9	72,90	30,8	125,00
Mato Grosso do Sul	53,4	86,60	26,6	160,00
Mato Grosso	51,4	87,00	26,5	163,00
Sudeste	48,2	89,50	26,7	153,00
Espírito Santo	51,3	71,20	26,9	120,00
Minas Gerais	55,3	70,60	31,6	118,00
Rio de Janeiro	47,9	100,20	32,0	127,00
São Paulo	38,6	115,40	19,6	206,00
Sul	36,2	137,40	16,3	361,00
Paraná	47,5	81,00	20,4	187,00
Rio Grande do Sul	33,2	161,00	15,8	462,00
Santa Catarina	26,1	173,20	11,8	426,00

UF: Unidade da Federação.

Nota: a tabela mostra a transferência média por beneficiário e a proporção média da população dos municípios que estaria coberta para cada região e UF, considerando desenhos alternativos de programas municipais de renda básica. O modelo “priorizando maximizar a cobertura” considera que os municípios dedicam uma parcela das suas despesas sociais igual à de Maricá ao programa (11,2%) e todos os indivíduos em famílias com renda até três salários mínimos são elegíveis. O modelo “priorizando focalização” considera que os municípios dedicam uma parcela das suas despesas sociais igual à de Niterói ao programa (9,2%) e todos os indivíduos em famílias pobres ou extremamente pobres são elegíveis.

Os resultados sugerem duas mensagens principais. A primeira é que, aos níveis de esforço fiscal considerados, há um *trade-off* entre cobertura e valor das transferências por beneficiários. Esse *trade-off* se acentua em regiões onde a despesa social é proporcionalmente maior e a proporção de pessoas em situação de vulnerabilidade social é menor. No Nordeste, programas municipais priorizando maximizar a cobertura cobririam, em média, 20% mais da população dos municípios do que programas priorizando a focalização, e as transferências por beneficiário seriam pouco mais do que 10% menores (R$ 5,30 menos por beneficiário). No Sul, programas priorizando maximizar a cobertura também cobririam, em média, 20% mais da população do que programas mais focalizados, mas as transferências por beneficiário seriam mais do que 60% menores (R$ 224,00 menos por beneficiário).

A segunda mensagem é que as desigualdades regionais entre programas de transferências municipais que demandassem parcelas similares à despesa social de todos os municípios seriam enormes. No modelo que priorizasse maximizar a cobertura do programa dentro dos municípios, as transferências por beneficiário seriam menos de um terço no Acre (R$ 33,30), estado com o menor valor, do que em Santa Catarina (R$ 173,20), estado com o maior valor. No modelo que priorizasse a focalização dos programas em indivíduos de famílias socialmente vulneráveis, as transferências por beneficiário seriam 13 vezes menores no estado com o menor valor (Acre, R$ 34,00) do que no estado com o maior valor (Rio Grande do Sul, R$ 462,00).

## Discussão

Neste estudo, investigamos a viabilidade de estender programas municipais de renda básica no Brasil, inspirados nos modelos do RBC-Maricá e do MSA-Niterói, analisando seu impacto nas finanças municipais. Segundo os nossos resultados, a implantação desses programas seria significativamente mais onerosa para a maioria dos municípios brasileiros do que para Maricá e Niterói. Em média, um programa nos moldes do RBC-Maricá demandaria 26,9% das despesas totais dos municípios e 38,7% de suas despesas sociais. Para o MSA-Niterói, esses percentuais seriam de 12,9% e 15,2%, respectivamente. Esses valores são substancialmente mais altos do que os observados em Maricá e em Niterói, que mantêm seus programas com menos de 5% das despesas totais e entre 9% e 12% das despesas sociais.

Além disso, os resultados sugerem a existência de marcadas desigualdades regionais na capacidade de implantação desses programas. Enquanto o MSA-Niterói poderia ser implantado pela maioria dos municípios da Região Sul com um esforço fiscal similar ao de Niterói, outras regiões, como Norte e Nordeste, enfrentariam um ônus fiscal excessivo. A implantação do RBC-Maricá se mostrou muito onerosa para a maioria dos municípios em todas as regiões - embora existam vários municípios no Sul e no Sudeste que poderiam arcar com os custos se fizessem um esforço similar ao de Maricá. Nesse sentido, é importante ressaltar que os resultados não reportados indicam que são justamente os municípios de maior renda em cada região os que teriam condições de implantar esses programas. Por exemplo, a renda domiciliar *per capita* média nos municípios da Região Sul onde o RBC-Maricá demandaria até 5% da despesa total é 25% superior à média regional. No Sudeste, essa diferença é de 65%. Para o modelo MSA-Niterói, nos municípios do Sul onde o programa demandaria até 5% da despesa, a renda média é 9% maior do que a média regional e, no Sudeste, 19% maior.

Ao explorar cenários onde os municípios fizessem um esforço fiscal similar ao de Maricá e de Niterói, observamos um *trade-off* inerente entre a cobertura do programa e o valor das transferências por beneficiário. Programas mais universalistas em regiões como o Nordeste poderiam cobrir uma parcela maior da população com uma pequena redução no valor do benefício, e no Sul, um aumento similar na cobertura resultaria em uma redução drástica nos valores transferidos. As transferências por beneficiário também seriam extremamente desiguais entre as UF, variando significativamente entre os modelos de programa que privilegiam a universalização ou a focalização. Essas diferenças nos valores provavelmente não seriam compensadas por diferenças no custo de vida entre as regiões. Por exemplo, na Região Norte o valor por beneficiário de programas de renda básica se os municípios fizessem esforços fiscais similares aos de Maricá ou de Niterói seria entre R$ 48,10 e R$ 64,00. No Sul, entre R$ 137,40 e R$ 361,00. Com a ressalva de que nós não temos dados sobre custo de vida para a maior parte dos municípios, essas diferenças são muito mais elevadas do que a diferença no custo de vida entre Belém (Pará) e Curitiba (Paraná) ou Porto Alegre (Rio Grande do Sul), que varia entre 10% e 15% ^30^.

Nossos achados corroboram a literatura que discute os desafios fiscais na implantação de programas de renda básica ^31^ ^,^ ^32^ ^,^ ^33^. A grande disparidade de custos entre os municípios brasileiros, especialmente quando comparados a Maricá e Niterói, que têm receitas atípicas de *royalties* de petróleo, ressalta a complexidade de transpor modelos bem-sucedidos em contextos específicos para uma realidade nacional heterogênea. A dependência de fontes de financiamento específicas, como *royalties*, torna a expansão nacional via recursos exclusivamente municipais uma tarefa árdua para a maioria dos entes federativos no Brasil.

Adicionalmente, os resultados sobre o *trade-off* observados entre cobertura e montante das transferências levantam questões cruciais sobre o desenho ideal de políticas públicas. A tensão entre o alcance e a suficiência do benefício, especialmente em cenários de recursos limitados, sugere que privilegiar maximizar a cobertura, se aproximando da universalização como idealizada pela *Lei nº 10.835/2004*, pode exigir um modelo de financiamento e implantação que transcenda a esfera municipal, considerando a ampla variação de capacidades fiscais e necessidades sociais entre os municípios brasileiros.

As profundas desigualdades regionais e o elevado custo fiscal para a maioria dos municípios indicam que a extensão de programas de renda básica no Brasil, exclusivamente com base em recursos municipais, é pouco provável. O financiamento com recursos municipais tende a favorecer os municípios mais ricos e/ou com maior poder de arrecadação, aprofundando disparidades regionais em vez de mitigá-las. Para alcançar todos os municípios brasileiros, será fundamental discutir outras formas de financiamento, possivelmente com um apoio substancial do Governo Federal.

Um programa de renda básica em nível nacional com um desenho homogêneo poderia endereçar as disparidades regionais e garantir um piso de proteção social mais equitativo ao longo do território nacional. Além do Programa Bolsa Família, a experiência bem-sucedida da expansão da Estratégia Saúde da Família nos municípios brasileiros mostra a necessidade de impulsão e coordenação do Governo Federal para a implantação de reformas bem-sucedidas nas políticas de seguridade social no país ^34^ ^,^ ^35^. Esses programas também mostram que a descentralização administrativa e avanços em direção à universalização da seguridade social são compatíveis quando o Governo Federal é um ator central do processo.

No entanto, as possibilidades de implantar um programa federal de renda básica em um contexto de restrições orçamentárias não parecem promissoras. O Governo Federal já financia programas de transferência de renda, como o Programa Bolsa Família e o BPC, além de repasses aos municípios vinculados com o financiamento do piso salarial dos agentes comunitários e enfermeiros, e outras políticas de saúde e educação. Adicionalmente, os poderes judiciário e legislativo têm aumentado o seu controle sobre parcelas do orçamento em anos recentes e o sistema tributário brasileiro continua sendo regressivo. Nesse sentido, a discussão sobre a expansão nacional de programas de renda básica não pode estar dissociada do debate sobre justiça fiscal e redistribuição de renda no Brasil, do qual as discussões sobre financiamento do estado de bem-estar social no país não podem estar escindidas. Em ausência dessa discussão, arrisca-se a defender versões de renda básica implantadas como um mecanismo de substituição e desmantelamento de outras intervenções do estado de bem-estar social, sendo que uma visão progressista da renda básica deveria defender que ela funcione como um complemento, e não um substituto, aos serviços públicos universais e outras garantias sociais ^36^.

A escolha do desenho do programa também se mostra crucial. Para um dado nível de esforço fiscal, há um *trade-off* entre a cobertura do programa e o montante das transferências por beneficiário. A definição do “melhor” desenho não é óbvia e dependerá dos objetivos prioritários da política pública: maximizar o alcance para todos os cidadãos, mesmo com benefícios menores, ou focar um valor mais substancial para os mais vulneráveis. De qualquer jeito, é necessário lembrar que a focalização excessiva é frequentemente contraproducente ^3^. O centro da questão é, então, como evitar as ineficiências associadas à focalização excessiva e garantir o tratamento uniforme de todos os cidadãos em condições de receber as transferências - criando mecanismos para a transição entre condições de elegibilidade e de inelegibilidade que sejam coerentes com o objetivo de universalizar certo nível de proteção social para todos ^37^.

É importante reconhecer algumas limitações inerentes a este estudo. Primeiro, apesar de pouco provável, o cálculo do MSA-Niterói pode ter levado a uma superestimação do valor médio das transferências em municípios com poucas famílias vulneráveis, mas com grande número de membros. Isso ocorre porque o cálculo assume uma distribuição uniforme de indivíduos em situação de vulnerabilidade social entre as famílias vulneráveis, não sendo possível replicar o limite familiar de R$ 700,00 por família diretamente com os dados agregados ao nível municipal. Segundo, não foram considerados os custos administrativos de implantação e gerenciamento dos programas, já que focamos apenas nos custos das transferências diretas. Terceiro, as análises não consideraram como as diferenças no custo de vida entre os diferentes municípios podem afetar o poder de compra dos programas em cada cenário.

Apesar dessas limitações, em conjunto, nossos resultados indicam que a expansão de programas municipais de renda básica no Brasil, nos moldes do RBC-Maricá e do MSA-Niterói, enfrentaria barreiras fiscais consideráveis para a maioria dos municípios, acentuadas por profundas desigualdades regionais. Essas barreiras inviabilizam a expansão, município a município, dos programas de renda básica investigados. Para que a *Lei nº 10.835/2004* seja efetivamente implantada, é necessário que se discuta um modelo de financiamento que transcenda a esfera municipal, possivelmente com forte participação do Governo Federal. Essa discussão necessariamente deve estar atrelada ao debate público sobre justiça fiscal e redistribuição de renda no Brasil, do qual o debate sobre a universalização de programas de renda básica não pode estar escindido. Essa abordagem não só poderia viabilizar avanços em direção à universalização de programas de renda básica, mas também contribuiria para a existência de maior equidade na distribuição dos benefícios em todo o território nacional e, eventualmente, para o bem-estar e a saúde da população.

## Data Availability

As fontes de informação utilizadas no estudo estão indicadas no corpo do artigo.
